# Conservative Treatment of Isolated Dorsal Dislocations of Fourth and Fifth Carpometacarpal Joints: A Report of a Rare Case

**DOI:** 10.7759/cureus.35356

**Published:** 2023-02-23

**Authors:** Dimitrios Giotis, Nikolaos K Paschos, Sotiris Plakoutsis, Dimitrios Vardakas, Christos Konstantinidis

**Affiliations:** 1 Orthopedics, General Hospital of Ioannina "G. Hatzikosta", Ioannina, GRC; 2 Orthopedic Surgery, Massachusetts General Hospital, Harvard Medical School, Boston, USA

**Keywords:** treatment, conservative, joints, carpometacarpal, dislocations, dorsal

## Abstract

Carpometacarpal (CMC) dislocations without associated fractures of the adjacent bones are extremely infrequent injuries. Dorsal or volar dislocations occur after high-energy injuries and may lead to early post-traumatic arthritis and carpal instability. The purpose of this study was to present a case of dorsal dislocation of both the fourth and fifth CMC joints that were treated with closed reduction and casting. A 31-year-old man developed severe acute pain, functional limitation, and deformity of the wrist after falling from a height. The clinical examination revealed intense localized tenderness, swelling, and palpable prominence over the fourth and fifth metacarpals. Standard anteroposterior and lateral views demonstrated dislocations of the examined CMC joints without any accompanied fracture. The injury was treated with anatomic closed reduction and cast immobilization for overall five weeks followed by early mobilization. Twelve weeks after injury, the patient had regained grip strength, and six months post-traumatically he satisfactorily returned to his previous hard labor-intensive activities without any functional deficits or chronic pain. Conclusively, CMC dislocations can be treated conservatively in case of early diagnosis and stable anatomic closed reduction.

## Introduction

Carpometacarpal (CMC) dislocations without associated fractures of the metacarpals and/or carpal bones are very infrequent injuries, accounting for less than 1% of all hand trauma [1]. Cooper (&) Roux were the first who observed this disorder in the 19th century [2]. This injury may be referred to isolated or multiple carpometacarpal joints, mostly related to direct high-velocity hand trauma, such as falls from height or car accidents [3]. According to the direction of the applied force and the form of causative mechanism, the dislocation of the metacarpals is classified as dorsal, which is more common, and volar [4].

In cases of missed or delayed diagnosis, CMC dislocations can lead to early post-traumatic arthritis and wrist instability [5]. A certain degree of controversy exists in the management of these injuries that can be either conservatively treated with closed reduction and casting, or surgically with open reduction and internal fixation. Advocates of surgical treatment highlight the risk of subluxation that can persist with conservative management while other surgeons advocate for initial conservative treatment when these injuries are recognized within the first eight hours. The purpose of this study is to present a case of dorsal dislocations of both fourth and fifth carpometacarpal joints which were treated successfully with closed reduction and casting and to highlight a possible early return to heavy pre-injury activities after conservative management.

## Case presentation

We report the case of a 31-year-old, right-hand-dominant man working at a mining factory and with no significant past medical history, who presented at the emergency department with severe acute pain after falling from a 1.5 meter-high platform onto his outstretched hand. He was assessed with the visual analog scale (VAS) for pain, and observed to have functional limitation and deformity of the wrist. The clinical examination revealed intense localized tenderness, swelling, and palpable prominence over the fourth and fifth metacarpals. Distal sensation and vascular function were intact. The passive range of motion (ROM) of the ring and little finger was painful whilst active ROM was nearly fully limited. 

**Figure 1:**
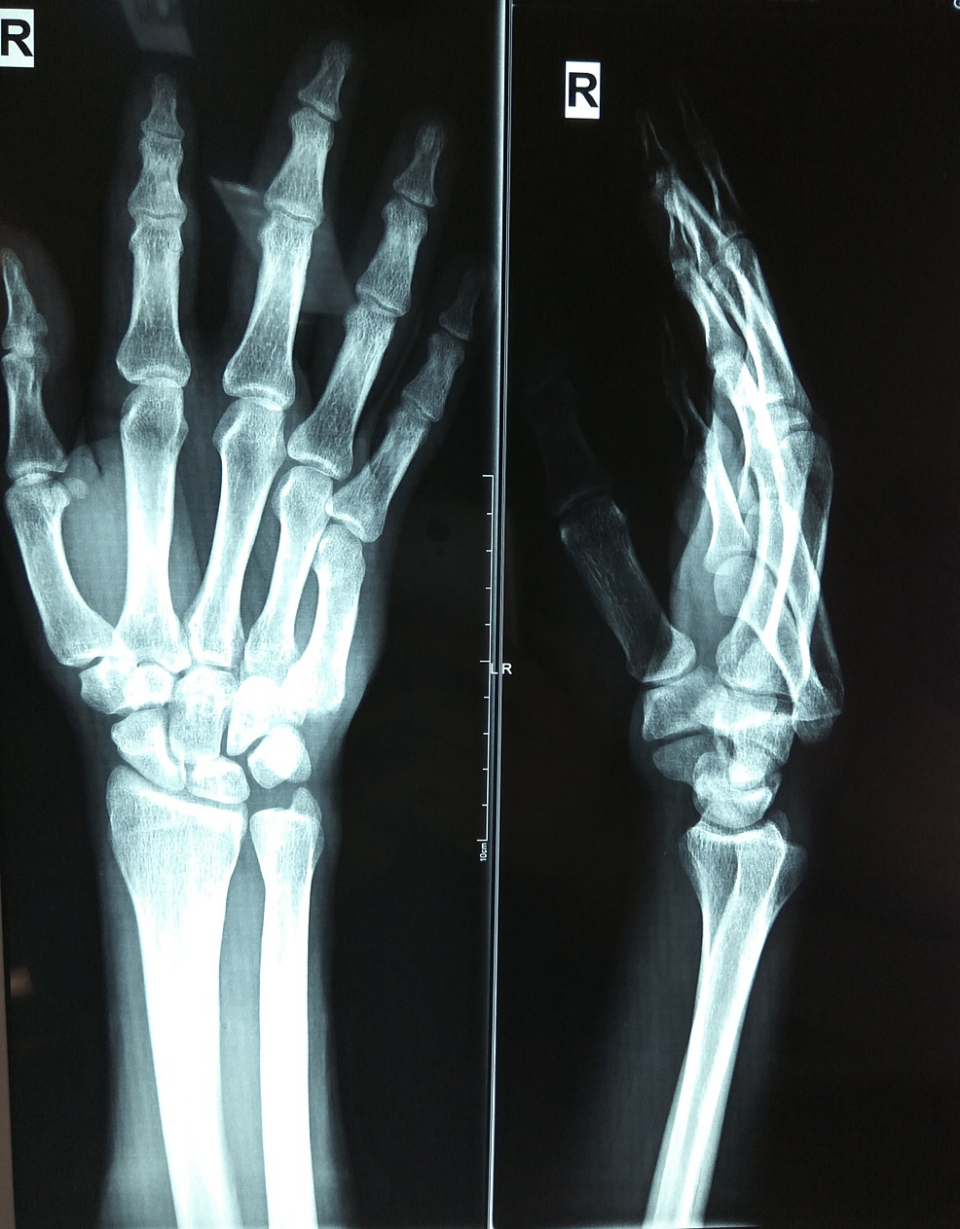
X-ray of the injured hand The anteroposterior and lateral views depict the isolated dorsal dislocations of both the fourth and fifth carpometacarpal joints

**Figure 2:**
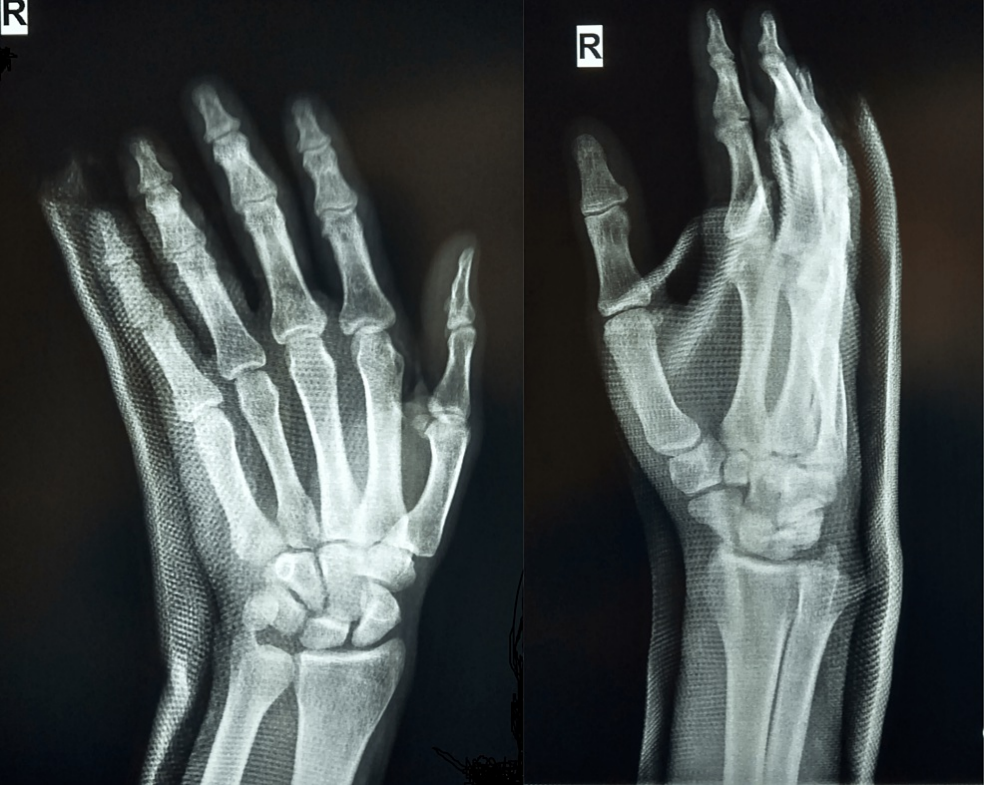
Radiograph (AP/lateral view) after the closed reduction and stabilization with ulnodorsal splint AP: Anteroposterior

Standard anteroposterior and lateral X-rays displayed a complete dislocation of the fourth and fifth CMC joints without any accompanied fracture (Figure 1). It was decided to reduce the dislocation instantly under regional anesthesia by plainly applying concurrent longitudinal traction and direct manual pressure on the dorsal base of the affected metacarpals. After the anatomic closed reduction was confirmed by new X-ray control (Figure 2), the congruity of all CMC joints was restored.

**Figure 3:**
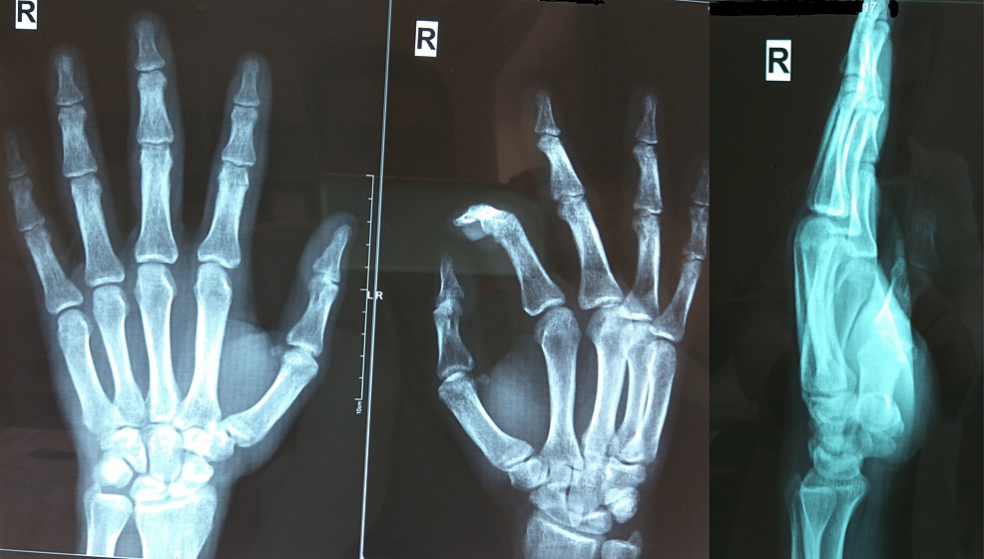
Anteroposterior, oblique, and lateral radiographic views two months after trauma

The reduction was relatively stable with no clinical sign of dorsal subluxation. Therefore, there was no need for surgical stabilization. An ulnodorsal splint extending from the metacarpophalangeal joints to the mid-forearm was applied to immobilize the two dislocated CMC joints for one week initially due to edema. Afterward, the splint was converted to a circular cast which was applied for a further four weeks. An X-ray control was performed every week to check the reduction. After cast removal, clinically the patient was free of pain and swelling, without any wrist instability. Final radiographs showed maintenance of normal anatomic reduction (Figure 3). 

The rehabilitation protocol which started five weeks post-traumatically comprised physical therapy with progressive active and passive mobilization of wrist and fingers, and muscles-tendons strengthening. Twelve weeks after injury, the patient had regained full painless ROM and grip strength returning to pro-injury daily activities. At the six-month follow-up, he had satisfactorily returned to his previous hard-labor activities without any functional deficits, discomfort, or chronic pain. Indeed, the active ROM was equal to his uninjured contralateral side, whilst grip strength was also equal bilaterally as evaluated by the Mayo Wrist Score (MWS). The MWS score was measured at 100 at the six-month follow-up and remained unalterable one year after injury.

## Discussion

Carpometacarpal joint dislocations are very rare injuries that are mainly associated with fractures of the adjacent bones [1]. Isolated CMC dislocations are even infrequent and mostly regard the fourth and fifth metacarpals [6]. The majority of them, approximately 70%, are dorsal [6]. Normally, the combined fourth and fifth CMC joint dislocations are identified in X-rays, due to loss of parallelism, asymmetry, and bone overlapping [6].

However, in the literature there have been reported cases of missed or delayed diagnoses, especially in patients with other obvious injuries, or when there are existing fractures in carpal/hand bones or extended diffuse hand swelling obscuring joint deformity that might mislead the clinicians, or even due to technically insufficient initial radiographs [7-10].

Clinical findings, such as acute pain, localized swelling of the dorsum of the hand and tenderness over the traumatized area, and axial deformity of the involved fingers with apparent shortening should always lead the clinician to take into consideration the likelihood of CMC joint dislocation [9]. Standard anteroposterior and lateral radiographs might be inadequate particularly when the metacarpal displacement is overlapped by other metacarpals [4]. Therefore, oblique views or even special radiographs should be included in X-ray control when standard views are unhelpful [6, 11,12]. When in doubt, further examination with CT would be definitive [13].

Left untreated or treated with delay, CMC dislocations whether dorsal or volar might predispose to severe complications which initially include carpal instability, swelling, residual pain, limited ROM, and reduced grip strength leading to early joint degeneration and progressively to post-traumatic arthritis causing severe morbidity [4,5,13,14]. Additionally, nerve dysfunction mainly due to edema can also be observed as the deep motor branch of the ulnar nerve is prone to damage after both dorsal and volar CMC dislocations of the fourth and fifth fingers [15,16].

After reviewing the literature which consists of almost solely sporadic case reports, there have been described cases of dorsal or volar CMC dislocations that were treated successfully with closed reduction and splint immobilization [17-19]. However, many authors advocate open reduction and internal fixation or closed reduction and percutaneous pinning to restore and maintain an anatomic reduction, and to prevent secondary dislocation [11,13]. Although there are no studies with a remarkable number of patients to extract safe conclusions for the treatment of these disorders, it is strongly recommended that in cases of associated fractures, delayed diagnosis, and insufficient or failed closed reduction, surgical intervention is the best management [4,7,8,10-12,14,16-18].

The most critical point for successful conservative treatment of CMC joint dislocations is the early and precise diagnosis along with an anatomic and stable closed reduction [17,18]. Due to the risk of secondary dislocations, especially within the first two weeks from injury, serial X-rays and close follow-ups should be always performed to avoid loss of reduction [17,20]. In such incidents, surgical treatment would be inevitable [1,17].

In our case, we report satisfactory results after a six-month follow-up in a young man who sustained dorsal dislocations of the fourth and fifth CMCs and was treated successfully with closed reduction and casting. The most important matter that led us to decide on conservative treatment was the early diagnosis and anatomic reduction that had been achieved. The weekly radiographic control allowed us to ascertain the maintenance of reduction. The patient who was dealing with hard manual work managed to return without functional limitation to his pre-injury work activities following an accelerated rehabilitation program. 

Similar results are reported by Agarwal (&) Agarwal in a patient who was treated conservatively for a divergent CMC dislocation of all four digits [18]. It was demonstrated that the patient managed to return to daily activities as a farmer despite residual hand swelling. On the other hand, Tingart et al. recommended operative therapy in patients who follow heavy manual work due to the increased risk of persisting pain [12].

## Conclusions

In conclusion, isolated dislocations of CMC joints can be treated successfully with closed reduction and casting as long as the recognition is accurate and not delayed. Moreover, an anatomical and stable reduction is another condition that plays an important role in the final clinical outcome, particularly for patients who take part in heavy manual daily activities.
